# Functional regions of the N-terminal domain of the antiterminator RfaH

**DOI:** 10.1111/j.1365-2958.2010.07056.x

**Published:** 2010-04

**Authors:** Georgiy A Belogurov, Anastasia Sevostyanova, Vladimir Svetlov, Irina Artsimovitch

**Affiliations:** Department of Microbiology and The RNA Group, The Ohio State University484 West 12th Avenue, Columbus, OH 43210, USA

## Abstract

RfaH is a bacterial elongation factor that increases expression of distal genes in several long, horizontally acquired operons. RfaH is recruited to the transcription complex during RNA chain elongation through specific interactions with a DNA element called *ops*. Following recruitment, RfaH remains bound to RNA polymerase (RNAP) and acts as an antiterminator by reducing RNAP pausing and termination at some factor-independent and Rho-dependent signals. RfaH consists of two domains connected by a flexible linker. The N-terminal RfaH domain (RfaH^N^) recognizes the *ops* element, binds to the RNAP and reduces pausing and termination *in vitro*. Functional analysis of single substitutions in this domain reported here suggests that three separate RfaH^N^ regions mediate these functions. We propose that a polar patch on one side of RfaH^N^ interacts with the non-template DNA strand during recruitment, whereas a hydrophobic surface on the opposite side of RfaH^N^ remains bound to the β′ subunit clamp helices domain throughout transcription of the entire operon. The third region is apparently dispensable for RfaH binding to the transcription complex but is required for the antitermination modification of RNAP.

## Introduction

RfaH is an operon-specific paralogue of the widely conserved general elongation factor NusG. NusG is essential in wild-type *Escherichia coli* (Sullivan and Gottesman, 1992) and is associated with RNAP transcribing most of the *E. coli* MG1655 genes (Mooney *et al.*, 2009a). Recent studies (Cardinale *et al.*, 2008) demonstrate that NusG becomes dispensable when the *rac* prophage *kil* gene is deleted and suggest that NusG limits transcription of the horizontally transferred DNA by enhancing Rho-dependent termination (Sullivan and Gottesman, 1992; Burns and Richardson, 1995). Additionally, *E. coli* NusG cooperates with NusA, NusB, NusE and other factors to form specialized anti-termination complexes that are resistant to pause and termination signals (Zhou *et al.*, 2002; Torres *et al.*, 2004).

By contrast, RfaH appears to act independently of other proteins and targets only those operons that have a 12 nt *ops* (operon polarity suppressor) element in their untranslated leader regions (Belogurov *et al.*, 2009). The *ops* element is required for RfaH recruitment to RNAP (Bailey *et al.*, 1997; Artsimovitch and Landick, 2002; Belogurov *et al.*, 2009); it mediates sequence-specific binding of RfaH to the non-template (NT) DNA strand exposed on the surface of the transcription elongation complex (TEC) and may induce TEC isomerization into a distinct state necessary for recruitment of RfaH. Our recent analysis identified several *ops*-containing operons that are enriched for RfaH (Belogurov *et al.*, 2009) in MG1655; these operons are devoid of NusG and do not encode essential functions. Consistently, RfaH is dispensable for growth of the commensal *E. coli* (Farewell *et al.*, 1991). However, RfaH activates expression of several virulence and fitness genes, such as LPS (Wandersman and Letoffe, 1993), capsule (Stevens *et al.*, 1994; Rahn and Whitfield, 2003) and haemolysin (Bailey *et al.*, 1992; Leeds and Welch, 1996) biosynthesis genes, and is essential for virulence (Nagy *et al.*, 2002; 2006;).

RfaH and NusG increase the transcript elongation rate and suppress RNAP pausing at backtracked sites *in vitro* (Artsimovitch and Landick, 2000; 2002;). However, some of their effects are different even in a purified system: for example, RfaH also reduces pausing at hairpin-dependent sites, whereas NusG does not. Most importantly, NusG increases, whereas RfaH reduces Rho-dependent termination (Artsimovitch and Landick, 2002; Mooney *et al.*, 2009b); this difference underlies the opposite regulatory functions of RfaH and NusG in the cell. RfaH inhibits Rho action and thus activates expression of laterally acquired genes (Belogurov *et al.*, 2009), whereas NusG appears to act in concert with Rho to inhibit expression of foreign genes (Cardinale *et al.*, 2008).

**Fig. 1:**
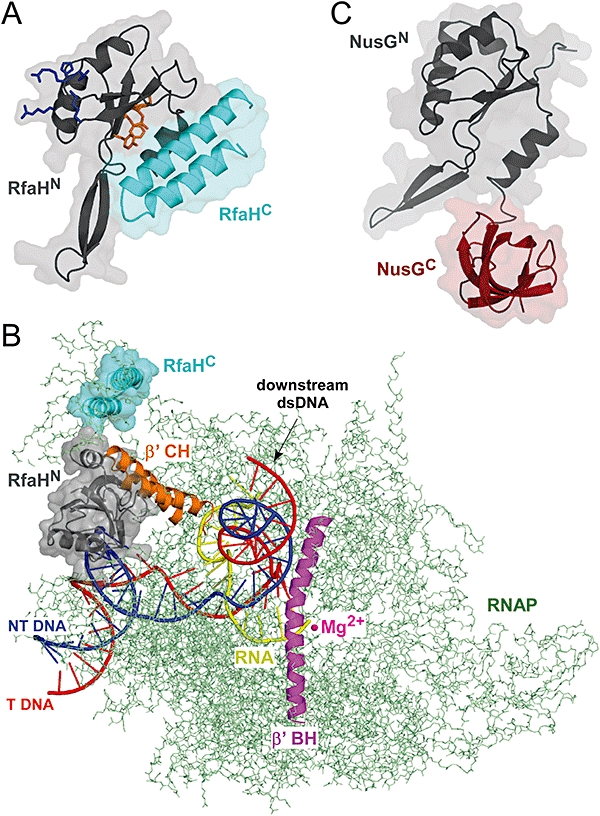
The structural context of the RfaH action. A. The *E. coli* RfaH model (Belogurov *et al.*, 2007) with RfaH^N^ shown in grey and the RfaH^C^– in cyan. The aromatic residues at the domain interface (orange) and the polar residues on the opposite side of the RfaH^N^ domain (blue) are shown as sticks. B. A model of RfaH bound to the TEC. The *T. thermophilus* RNAP (Vassylyev *et al.*, 2002) is shown as green lines with the bridge helix (β′ BH) highlighted in magenta, the template DNA – in red, the NT DNA – in blue, the nascent RNA – in yellow. Position of the RNAP active site is marked by the high-affinity catalytic Mg^2+^ ion (a small magenta sphere). RfaH^N^ (grey) is bound to the NT DNA strand and to β′ CH, whereas RfaH^C^ (cyan) makes no contacts to RNAP. C. The *E. coli* NusG model (Steiner *et al.*, 2002) is shown for comparison, the structurally distinct NusG^C^ domain is shown in dark red.

These opposite functions may be partially explained by different architectures of the two proteins (Fig. 1). Both proteins consist of two domains connected by a flexible linker (Belogurov *et al.*, 2007). The N-terminal domains (RfaH^N^ and NusG^N^) are structurally similar and mediate RNAP binding and anti-pausing (AP) activities of both proteins (Belogurov *et al.*, 2007; Mooney *et al.*, 2009b). The C-terminal domains are drastically different (Fig. 1A and C): a short α-helical hairpin in RfaH, a β-barrel Tudor domain in NusG. Strikingly, the RfaH^C^ sequence can be computationally fitted into a NusG^C^-like structure (Belogurov *et al.*, 2009). The two domains are tightly associated in a free RfaH, and the interdomain interface masks a hydrophobic surface on RfaH^N^ that is thought to serve as an RNAP binding site; we have proposed that RfaH binding to an *ops* element triggers the domain separation and allows RfaH binding to the RNAP (Belogurov *et al.*, 2007). In contrast, the two NusG domains do not interact, implying that the RNAP-binding surface on NusG^N^ is always accessible (Mooney *et al.*, 2009b); indeed, NusG associates with most transcribed operons (Mooney *et al.*, 2009a).

In a model of RfaH bound to the TEC, one side of RfaH^N^ binds to the exposed segment of the NT stand and the other to the tip of the β′ clamp helices (CH) domain, whereas the RfaH^C^ domain does not make any contacts to RNAP or DNA (Fig. 1B). This model is supported by (i) zero-length UV-cross-linking of RfaH to the NT DNA (Artsimovitch and Landick, 2002); (ii) the ability of RfaH^N^ to compete with σ^70^ for binding to the β′ CH during elongation (Sevostyanova *et al.*, 2008); (iii) the loss of *ops* binding conferred by substitutions of adjacent basic residues in RfaH (see *Results*); (iv) the deleterious effects of substitutions of the hydrophobic residues in RfaH and in the β′ CH on RfaH association with the TEC (Belogurov *et al.*, 2007; Sevostyanova *et al.*, 2008).

Here, we show that RfaH^N^ also supports anti-termination at Rho-dependent and intrinsic terminators *in vitro*. Taken together with our previous reports that RfaH^N^ is sufficient for the AP activity and σ^70^ exclusion *in vitro* (Svetlov *et al.*, 2007; Sevostyanova *et al.*, 2008), these data indicate that RfaH^N^ contains all the elements required for RfaH function. In this work, we set out to dissect these elements. We constructed a set of single residue substitutions in RfaH^N^ and tested their phenotypes *in vitro*. We report that the two previously hypothesized *ops*-binding and RNAP-binding regions (Fig. 1A) are indeed required for RfaH recruitment to the TEC. Unexpectedly, our analysis also identified a third RfaH^N^ cluster, which is apparently dispensable for binding to the TEC but is essential for the AP activity of RfaH.

## Results

### RfaH^N^ mediates all transcriptional activities of RfaH *in vitro*

We reported that RfaH^N^ is sufficient for the AP activity of RfaH at factor-independent pause signals (Belogurov *et al.*, 2007) and at σ-dependent pause sites (Sevostyanova *et al.*, 2008). Similarly, Mooney *et al.* (2009b) have found that NusG^N^ is sufficient for NusG's effects on RNA chain elongation. However, NusG^N^ does not support increased Rho-dependent termination. Thus, we wanted to test whether RfaH^N^ alone can mediate the effects of full-length RfaH on Rho-dependent termination. RfaH^N^, which possesses an extensive hydrophobic surface that is masked either by RfaH^C^ (in a free RfaH) or by the β′ CH (when bound to the TEC), is insoluble when overexpressed separately. To circumvent this problem, we introduced a TEV protease cleavage site into the interdomain linker of the C-terminally His-tagged RfaH, cleaved the purified full-length protein using the His-tagged TEV protease, and removed both the protease and the RfaH^C^ domain by absorption to the Ni-Sepharose resin. Thus, isolated RfaH^N^ is poorly soluble and prone to aggregation but, when present at low concentration, acts similarly to the full-length RfaH (Belogurov *et al.*, 2007).

**Fig. 2:**
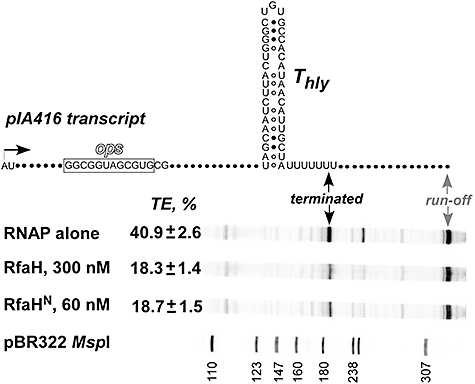
RfaH^N^ effects on intrinsic termination. Transcript generated on a linear pIA416 DNA template; transcription start site (+1), the *ops* element (boxed), T*_hly_* terminator structure, terminated and run-off RNA products are shown on top. Halted [α-^32^P]-CMP-labelled G37 TECs were formed at 60 nM with *E. coli* RNAP and challenged with NTPs (10 µM UTP, 200 µM ATP, CTP, GTP) and rifapentin at 25 µg ml^−1^ in the absence or in the presence of full-length RfaH or RfaH^N^. The reactions were incubated for 15 min at 37°C, quenched, and analysed on a 6% denaturing gel along with the [γ-^32^P]-ATP-labelled pBR322 *Msp*I digest as a molecular weight standard (the sizes of fragments are indicated. Termination efficiency (219-nt long RNA as a fraction of total RNA) was determined in three independent experiments.

We tested the effect of RfaH^N^ at the intrinsic (factor-independent) T_hly_ terminator, which has been shown to respond to RfaH *in vivo* (Koronakis *et al.*, 1988) and *in vitro* (Carter *et al.*, 2004). During single-round *in vitro* transcription in the absence of RfaH, ∼40% of transcripts were terminated at T*_hly_* (Fig. 2), whereas addition of RfaH decreased termination efficiency more than twofold, to 18%; the same effect has been reported previously (Carter *et al.*, 2004). Consistent with its key role in modification of RNAP into a pause-resistant state, the isolated RfaH^N^ domain had the same effect on termination at T*_hly_* (18%). An apparent reduction in an overall level of RNA synthesis observed in the presence of RfaH or RfaH^N^ is due to the RNAP arrest at the *ops* site under these conditions (not visible on the gel) and does not affect data interpretation: those RNAP molecules that do reach the termination site bypass it more efficiently than in the absence of RfaH.

**Fig. 3:**
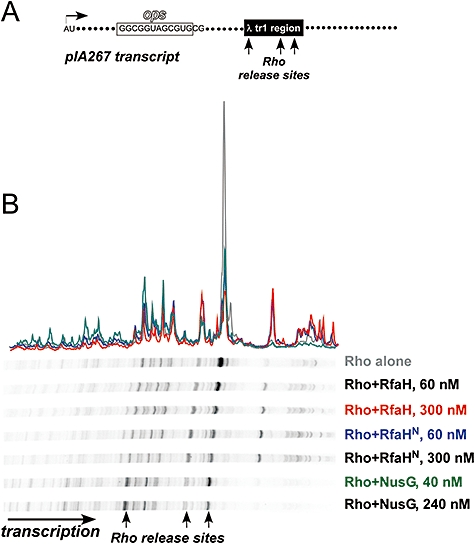
RfaH^N^ effects on Rho-dependent termination. A. Transcript generated on a linear pIA267 DNA template; transcription start site (+1), *ops*, Rho-dependent RNA release sites and transcript end are indicated. B. Halted, [α-^32^P]-GMP-labelled TECs were formed at 40 nM with *E. coli* RNAP. Rho, NusG, RfaH or RfaH^N^ were added at indicated concentrations, followed by addition of NTPs and rifapentin. The reactions were incubated for 15 min at 37°C, quenched, and analysed on a 6% denaturing gel. A representative gel and four selected traces for Rho alone (gray), full-length RfaH at 300 nM (red), RfaH^N^ at 60 nM (blue) and NusG at 40 nM (green) are shown.

To assay RfaH^N^ effect on Rho-mediated RNA release, we used a template that encodes the *opsP* followed by a well-characterized phage λ_tR1_ Rho-dependent termination signal [Fig. 3A, pIA267 (Artsimovitch and Landick, 2002)]. On this template, RfaH and NusG had opposite effects on Rho-dependent RNA release: consistent with its synergy with Rho, NusG shifted the distribution of RNA species towards shorter transcripts, whereas RfaH favoured synthesis of longer RNAs (Fig. 3B), possibly by reducing RNAP pausing, and thus Rho-mediated termination. The RfaH^N^ domain displayed the same effect but was able to act at lower concentrations; the enhanced activity of RfaH^N^ was also observed in pause assays (Belogurov *et al.*, 2007) and may be due to the higher stability of RfaH^N^/TEC complex. We conclude that the isolated RfaH^N^ domain is sufficient for all documented *in vitro* activities of RfaH.

### *In vitro* assay for *ops* binding and AP activities of RfaH

**Fig. 4:**
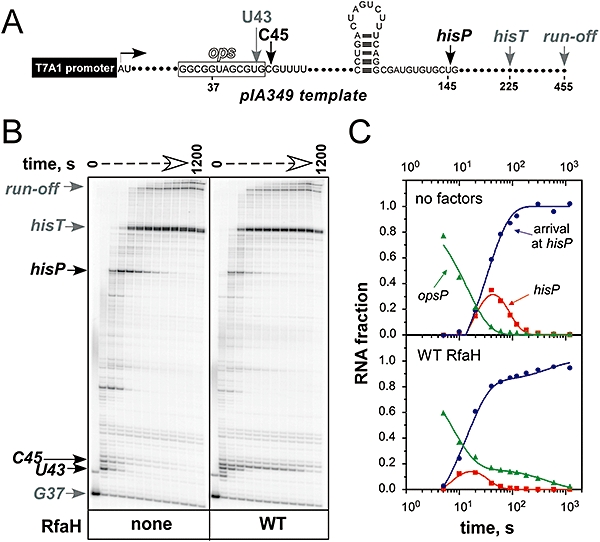
Effects of RfaH on transcription elongation *in vitro*. A. A schematic representation of a linear template pIA349 with the *ops* element, the start site (+1), transcript end (run-off), the pause sites that occur after the addition U43 (*opsP1*), C45 (*opsP2*) and U145 nucleotides (the *hisP* pause), and the *hisT* terminator indicated. B. WT RfaH accelerates TEC escape from the U43 and the *hisP* sites but delays the RNAP escape from the C45 site in a single-round elongation assay. Halted radiolabelled G37 TECs (see *Experimental procedures*) were pre-incubated with RfaH at 50 nM or storage buffer for 5 min at 37°C, and then challenged with rifapentin at 100 µg ml^−1^ and NTPs (10 µM GTP, 150 µM ATP, CTP, UTP). Aliquots were withdrawn at times ranging from 5 to 1200 s and analysed on a 8% denaturing gel. C. The fractions of RNA at the *hisP* (red squares), at or beyond the *hisP* site (blue circles), and at the *opsP* site (U43 + G44 + C45; green triangles) were quantified from the gel in (B) and used to kinetically model the pauses in the absence (top) and in the presence (bottom) of the WT RfaH.

RfaH has several distinct effects in an *in vitro* transcription assay: it delays RNAP escape from the *ops* signal, reduces pausing at hairpin-dependent (class I) pause sites and inhibits backtracking at class II sites (Artsimovitch and Landick, 2002; Svetlov *et al.*, 2007). To simultaneously assay the *ops* recognition and the AP activity of RfaH, we utilized a pIA349 template that encodes the tandem *ops* and *his* pause signals downstream from a T7A1 promoter (Fig. 4A). The initial transcribed region was designed to allow for the formation of radiolabelled TECs stalled after incorporation of a G residue at position 37 (G37) when transcription is initiated in the absence of UTP (Fig. 4A). Upon addition of all four NTP substrates and rifapentin (to block re-initiation), RNAP elongated the nascent RNA at a characteristic rate, pausing at *opsP1*, *opsP2* and *hisP* sites (Fig. 4B).

Here, we use the hairpin-dependent *hisP* signal to evaluate the AP activity of RfaH and its variants. The *hisP* signal is the best characterized model system for the analysis of pausing by the *E. coli* RNAP; the molecular mechanism of hairpin-induced pausing (Toulokhonov *et al.*, 2007) and its response to various regulatory factors, including RfaH (Artsimovitch and Landick, 2000; 2002;) have been characterized. The wild-type (WT) RfaH reduced pausing at the *hisP* site (position U145 in the pIA349 transcript; the red line in Fig. 4C), as well as other pause sites.

To evaluate the DNA-binding activity of RfaH variants, we measured RfaH-induced retention of RNAP at the *ops* signal. RfaH binds with high affinity (with *K*_d_ in a low nM range) to the TEC paused at the conserved *opsP1* site (Artsimovitch and Landick, 2002), which corresponds to position U43 in pIA349 transcript (Fig. 4A). During *in vitro* transcription (Fig. 4B), the WT RfaH delayed a small fraction of RNAP (∼15–20%) two nucleotides downstream of *opsP1* (e.g. *opsP2* site, position C45). The escape kinetics from the *ops* region (positions U43, G44 and C45, shown by the green line in Fig. 4C) became biphasic. The initial slope (largely attributed to *opsP1*) was the same as in the absence of RfaH (Fig. 4C, top). In contrast, the second, slower escape phase (attributed to *opsP2*) was only observed in the presence of RfaH. We argued that this delay is likely due to contacts of RfaH with the NT DNA that must be broken to allow RNAP escape from the *opsP* site (Artsimovitch and Landick, 2002).

Importantly, RfaH binds DNA only in the context of the *ops*-paused TEC; RfaH affinity for an *ops* DNA oligonucleotide is ∼1000 times lower than for the TEC. Modelling suggests that DNA must be deformed to allow for productive contacts with RfaH (Svetlov *et al.*, 2007); thus, both a specific conformation of the NT DNA on the RNAP surface and RfaH interactions with the RNAP surface residues may contribute to its increased affinity for the TEC. We cannot separately determine RfaH affinities for the *ops* DNA and RNAP by a conventional binding assay. Because RfaH remains stably bound to the TEC for the duration of the transcription cycle (Belogurov *et al.*, 2009), and therefore likely retains contacts with RNAP and non-specific interactions with DNA, we attribute the RNAP delay at *opsP2* primarily to the sequence-specific interactions between RfaH and the *ops* element, and use this delay as an indirect assay to evaluate the strength of interactions between RfaH and the NT DNA.

Experiments performed in the presence of GreB (Fig. S1) suggest that RNAP paused at *opsP2* is backtracked by two nucleotides, so that the active site is located at the *opsP1* site. RfaH therefore likely maintains specific contacts with the NT DNA while RNAP moves at least two nucleotides downstream. Structural and biochemical analyses suggest that the NT DNA segment exposed on the surface of the elongating RNAP is short (4–5 nucleotides) and constant at most sites and in TECs from diverse organisms (Korzheva *et al.*, 2000; Kettenberger *et al.*, 2004; Vassylyev *et al.*, 2007); however, no TEC structure with the transcription bubble intact is available, and thus the actual path of the single-stranded NT DNA on RNAP remains unknown. Based on structural modelling (Belogurov *et al.*, 2007), we proposed that four *ops* nucleotides at the upstream edge of the bubble may be involved in direct contacts with RfaH detected by cross-linking (Artsimovitch and Landick, 2002). RNAP translocation by two nucleotides following RfaH recruitment would occlude the RfaH binding site on the NT DNA unless moderate accumulation, or ‘scrunching’ (Cheetham and Steitz, 1999), of the DNA occurs within the transcription bubble (Fig. S1). The hypothetical scrunched state can be resolved either by breakage of the RfaH/NT DNA contacts or by reannealing of scrunched DNA. The latter scenario is phenotypically indistinguishable from backtracking to the recruitment site as observed on the pIA349 template (Fig. S1). The RfaH-induced delay is only observed when the third nucleotide downstream from *opsP1* is G and the GTP concentration is low (data not shown), indicating that this effect has little relevance to RfaH function *in vivo*.

The main goal of this work is to identify the RfaH determinants that mediate its recruitment to the TEC and the consequent AP modification of RNAP. We focused on mutational analysis of RfaH^N^, since it is responsible for all the direct effects of RfaH on transcript elongation and termination. We targeted both the surface-exposed residues that are highly conserved in the NusG-RfaH superfamily (e.g. a subset of hydrophobic residues that may contact the β′ CH) and those residues that are divergent between RfaH and NusG (e.g. the positively charged residues that may constitute the DNA binding site in RfaH). Below, we summarize the properties of selected single substitutions in RfaH at the *opsP2* and *hisP* sites.

### Substitutions that compromise RfaH-induced pausing at *ops*

In our analysis, we treated three consecutive positions within the *opsP* site (U43, G44 and C45) as a single pause site (See *Supporting information*). In the absence of RfaH, the majority of RNAP molecules occupied the *opsP1* site (U43) and escaped following a monoexponential function with a rate constant of ∼0.1 s^−1^. In the presence of WT RfaH, the U43 site became depopulated and the RNAP occupancy at G44 and C45 positions was increased; a slowly escaping fraction (∼17%, rate constant 0.003 s^−1^) emerged at C45, necessitating the use of a biexponential function for an accurate fit of escape kinetics.

**Fig. 5:**
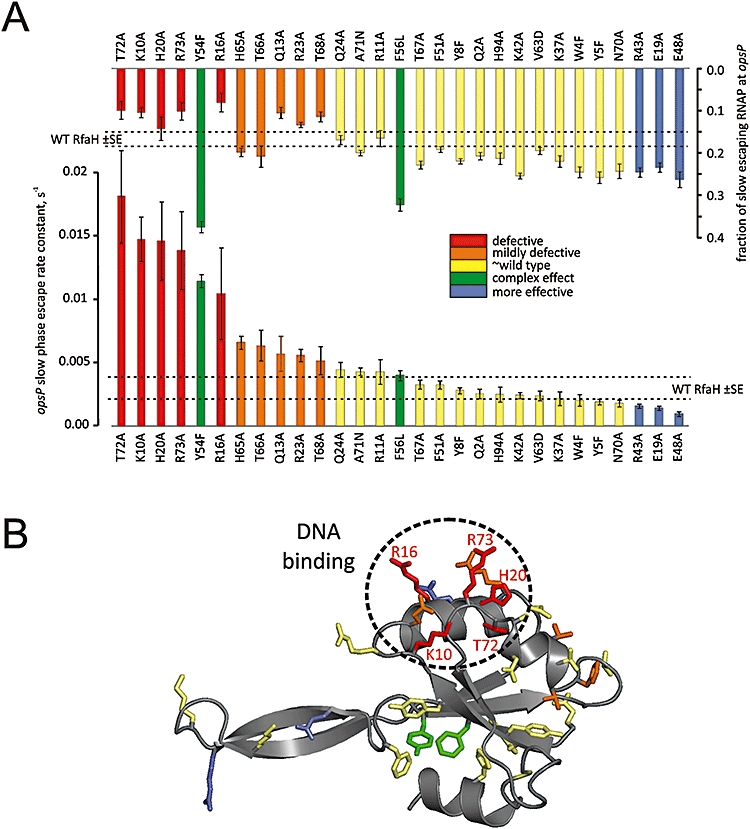
Effects of RfaH^N^ substitutions on pausing at the *ops* site. A. Effects of altered RfaH proteins on pause efficiency at (top) and escape rate from (bottom) the C45 position (Fig. 4). See text for details. B. Substitutions are shown on the RfaH^N^ structure as sticks and coloured according to their phenotypes (colour coded as in A). The position of a hypothetical DNA-binding region is indicated.

The effects of various RfaH substitutions on RNAP delay at C45 are summarized in Fig. 5. K10F and R73D variants were strongly defective at *opsP*: RNAP displayed monoexponential escape kinetics and the pausing pattern was indistinguishable from that observed in the absence of RfaH. Thus, these variants are absent from Fig. 5A. Several variants retained partial activity: they delayed 8–40% of RNAP molecules at C45. Five substitutions (T72A, K10A, H20A, R73A and R16A) led to strong defects; the escape rate constant increased three- to ninefold, whereas the fraction of delayed RNAP was similar or reduced relative to the WT RfaH. These residues form a tight cluster (Fig. 5B) that we propose constitutes the DNA binding site. Lys-10, Arg-16, His-20 and Arg-73 side-chains face the protein exterior and may make direct contact with the DNA. In contrast, Thr-72 faces the interior and therefore likely affects DNA binding indirectly, e.g. by controlling the position of Arg-73.

H65A, T66A, Q13A, R23A and T68A variants appeared mildly defective in NT DNA binding: the RNAP escape rate constant was increased 1.5- to 2-fold and the fraction of delayed RNAP was similar or decreased as compared with WT RfaH. Gln-13 and Arg-23 are adjacent to the residues forming a putative DNA-binding cluster and may either directly interact with DNA or affect positioning of the primary DNA-binding residues. On the other hand, we hypothesize that the effects of H65A, T66A and T68A substitutions are indirect and may result from compromised RfaH interactions with RNAP (see *Discussion*).

Three substitutions (E19A, R43A and E48A) were more effective than WT RfaH: they reduced the escape rate constant two- to threefold and increased the fraction of C45-delayed RNAP by ∼30%. The apparent stabilization of the RfaH-DNA contacts by E19A substitution may be due to the removal of the negatively charged Glu side-chain from the vicinity of the DNA-binding residues. Glu-48 may interact with the invariant Arg-178 from RfaH^C^ in free RfaH and with the β′ CH residues in the RfaH–TEC complex. Changes in both the interdomain and intermolecular contacts upon E48A substitution may contribute to the observed effect, because escape of at least some RNAP from the backtracked state at C45 may involve RfaH dissociation from the TEC. The effect of R43A substitution at the tip of a β-loop is difficult to interpret; this loop is predicted to be highly mobile even in RfaH that is not bound to RNAP (Fig. S2). The β-loop may be involved in interactions with the β′ CH or with the DNA fork junction and thus contribute to the RfaH ability to reduce backtracking (Svetlov *et al.*, 2007). In *E. coli* NusG, the size but not the sequence of this ‘mini-domain’ is important for activity (Richardson and Richardson, 2005).

Finally, we placed Y54F and F56L into a separate group since these variants delayed as much as 35–40% RNAP at C45 and were thus clearly distinct from the WT and all other variants. Tyr-54 is one of the most conserved residues in NusG and all of its paralogues; in contrast, Phe-56 is only conserved in RfaH-like proteins, whereas most NusGs have a Leu at this position (Belogurov *et al.*, 2009). The two substitutions also had different effects on escape rate, similar to WT for F56L and four times greater in the case of Y54F. Both Tyr-54 and Phe-56 are located on the RfaH^N^ surface thought to constitute the β′ CH binding site (Belogurov *et al.*, 2007). It is possible that their substitutions cause RfaH to bind RNAP in an aberrant way that increases the backtracking propensity of the RfaH–TEC complex at C45. The F56L protein, which mimics the NusG configuration, has a ‘wild-type’ effect on RNAP escape, implying that it remains tightly bound to the TEC. In contrast, the more facile RNAP escape from C45 observed in the presence of Y54F suggests that the altered protein readily dissociates from the backtracked complex, thereby allowing the TEC to translocate forward and resume elongation.

### Substitutions that compromise the AP activity of RfaH^N^

**Fig. 6:**
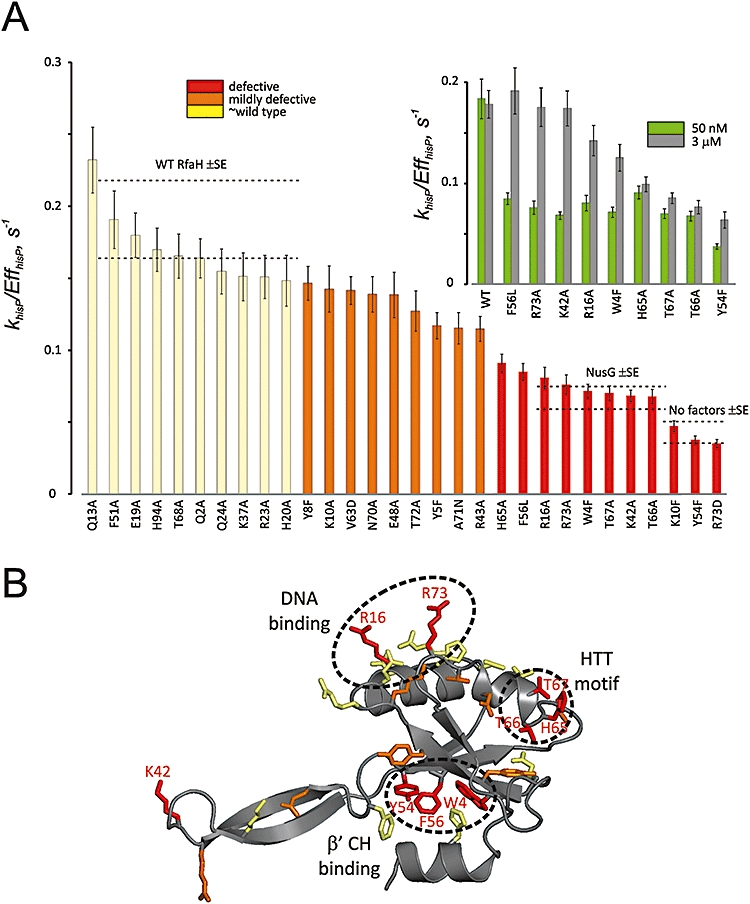
Effects of RfaH^N^ substitutions on pausing at the *hisP* site. A. Effects of altered RfaH proteins on pausing at *hisP* at 50 nM protein (Fig. 4). Inset; the AP activity measured at 3 µM RfaH. B. Substitutions are shown on the RfaH^N^ structure as sticks and coloured according to their phenotypes (colour coded as in A). The positions of a presumed β′ CH binding site and an HTT motif are indicated, together with that of the hypothetical DNA-binding region (Fig. 5B).

After RfaH is recruited to RNAP at the *ops* site, it remains bound to the enzyme for thousands of nucleotide addition cycles (Belogurov *et al.*, 2009), preventing NusG binding, inhibiting backtracking and reducing efficiency and longevity of all known types of pauses. Pausing at the *hisP* site is traditionally analysed by fitting the decay part of the pausing curve to a monoexponential function. This analysis outputs well-defined escape rate and pause efficiency (extrapolated to zero time) parameters but serves as a rough approximation of the actual events. Indeed, in our assays RNAP was starting to arrive at *hisP* only after 5–10 s and continued to simultaneously arrive and escape for tens or even hundreds of seconds. In addition, different RfaH mutants produced different proportions of fast and slow (*opsP2* delayed) fractions of RNAP and also generated various degrees of asynchrony in RNAP arrival to *hisP* site. We thus employed a more complex kinetic model that accounts for the concurrent arrival and escape of both the fast and the slow fractions of RNAP at *hisP* (see *Supporting information*). Our analysis demonstrates that only the ratio of escape rate constant and efficiency (*k_hisP_/Eff_hisP_*), but not the individual parameters, can be accurately determined for all datasets. This limitation is inherent in the experimental set-up (concurrent arrival and escape at *hisP* with similar rate constants) and is not due to excessive complexity of the model. We therefore used *k_hisP_/Eff_hisP_* as a measure of AP activity of RfaH variants (Fig. 6).

Q13A, F51A, E19A, H94A, T68A, Q2A, Q24A, K37A, R23A and H20A variants displayed *k_hisP_/Eff_hisP_* ratio indistinguishable from that of the WT RfaH within the margin of error, suggesting that the altered residues are not essential for TEC binding and AP activity. In contrast, H65A, F56L, R16A, R73A, W4F, T67A, K42A and T66A variants displayed *k_hisP_/Eff_hisP_* ratio similar to that observed with NusG and two- to threefold lower than that observed with the WT RfaH. K10F, Y54F and R73D were strongly defective: they did not elevate *k_hisP_/Eff_hisP_* ratio above that observed in the absence of added elongation factors. Finally, Y8F, K10A, V63D, N70A, E48A, T72A, Y5F, A71N and R43A variants displayed mild defects, with *k_hisP_/Eff_hisP_* ratio in the range of 80–60% of the WT RfaH value. We note that mildly defective variants cannot be unambiguously differentiated from the WT group due to a continuum in decreasing *k_hisP_/Eff_hisP_* values. The substitutions producing mild phenotypes are usually adjacent to those causing strong defects (Fig. 6B), suggesting that both types of substitutions interfere, directly or indirectly, with the same interaction in the RfaH–TEC complex.

We then focused on pinpointing the causes of diminished AP activity in strongly defective variants. The diminished activity may arise from (i) inefficient recruitment at *opsP*, (ii) dissociation of RfaH prior to arrival to *hisP*, or (iii) failure of the TEC-bound RfaH to reduce pausing. At concentrations above 2 µM, RfaH does not require *opsP* for function *in vitro*, implying that RfaH can be recruited to RNAP non-specifically (data not shown). Thus, both the inefficient recruitment and the subsequent dissociation should be compensated by increasing the concentration of the RfaH variant. Indeed, at 3 µM, F56L, R73A and K42A displayed *k_hisP_/Eff_hisP_* ratios indistinguishable from that observed in the presence of WT RfaH (Fig. 6A, inset), whereas partial compensation (a 1.5-fold increase in *k_hisP_/Eff_hisP_* ratio) was observed with R16A and W4F. These results suggest that defects in recruitment to, or retention on, the TEC were the primary causes of diminished AP activity for W4F, R16A, K42A, F56L and R73A variants.

In contrast, the low *k_hisP_/Eff_hisP_* ratios of H65A, T67A and T66A were not markedly affected by a 60-fold increase in concentration, suggesting that these substitutions eliminated AP activity. This is supported by the observation that H65A, T67A and T66A variants remain tightly associated with the TEC (A. Sevostyanova *et al.*, manuscript in preparation). Finally, increasing Y54F RfaH concentration to 3 µM resulted in 1.5-fold higher AP activity, but the *k_hisP_/Eff_hisP_* ratio remained threefold below that of WT RfaH and equal to that of NusG. We conclude that the Y54F change not only compromises RfaH retention on the TEC, but likely also completely abolishes RfaH–TEC interactions essential for its AP activity at the *hisP* (which NusG lacks).

To assess the impact of substitutions on the RfaH structure, we first utilized a CONCOORD-PBSA molecular mechanics approach (Benedix *et al.*, 2009). The predicted effects of most substitutions on stability and overall structure were small (ΔΔG ± 2 kcal mol^−1^ and root mean square values < 2 Å relative to WT RfaH; Table S2), and did not correlate with a given mutant's AP activity, although variants with an increased stability tended to have increased or near-WT levels of activity. Alignment of the structural ensembles for each variant and the starting RfaH structure (PDB ID 2OUG) demonstrated that predicted changes in flexibility were insignificant in the case of the closed conformation, the state for which structural information exists (Figs S2 and S3). This suggests that the detrimental effects of substitutions analysed in this work are not mediated by gross changes in the RfaH structure or folding.

To verify the modelling predictions experimentally, we carried out CD analysis of selected RfaH variants (Fig. S4). This analysis revealed no gross structural alterations conferred by substitutions. In particular, the spectrum for the Y54F protein, which displays dramatic defects both *in vitro* (Figs 5 and 6) and *in vivo* (see the next section), was indistinguishable from that of WT RfaH.

### *In vivo* effects of RfaH^N^ substitutions

**Fig. 7:**
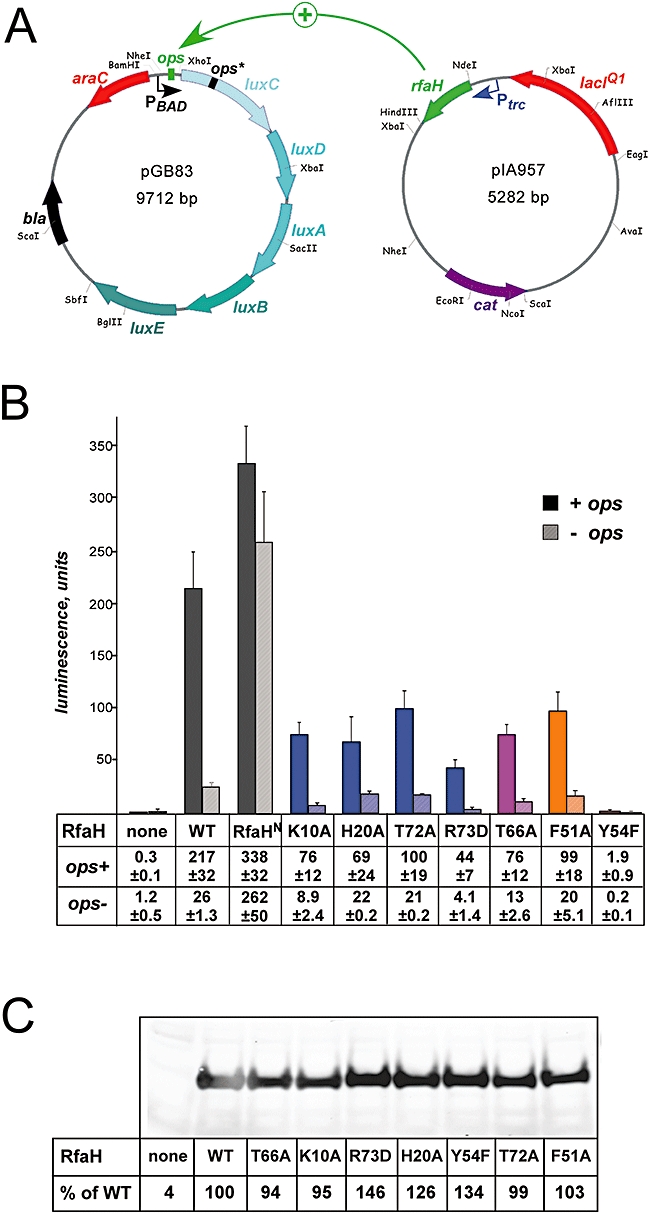
The *in vivo* reporter assay for the RfaH activity. A. The assay system. We constructed an *rfaH* knockout strain and two plasmids to assay the effects of *ops* and RfaH variants *in vivo*. The first plasmid (pGB83; left) has a *Col*E1 origin of replication and contains the entire *Photorhabdus luminescens* (Winson *et al.*, 1998) *luxCDABE* operon under the control of the AraC-controlled P_BAD_ promoter and an *ops* element. The second compatible (P15A *ori*) plasmid (right; pIA957) has the *E. coli rfaH* gene cloned under the P*_trc_* control; the plasmid also carries an engineered *lacI*^Q1^ gene. B. Analysis of the effects of selected RfaH substitutions on *lux* operon expression in the presence (dark bars) and the absence (light bars) of the *ops* element. The results are expressed as luminescence corrected for the cell densities of individual cultures divided by a factor of 1000. The data represent the average of at least four independent experiments. C. Western analysis of cell extracts expressing RfaH variants (as in panel B; see *Experimental procedures*) performed in parallel with the *lux* assay with polyclonal RfaH^C^-specific antibodies.

We wished to test whether the RfaH residues implicated by our *in vitro* assays are functionally important in the cell. We designed an assay system (Fig. 7A) consisting of three components. First, we constructed an *rfaH*^-^ strain by a targeted disruption of the *rfaH* gene in *E. coli* DH5α strain (see *Experimental procedures*). Second, we constructed a compatible low-copy-number plasmid (pIA957) with the *rfaH* gene cloned under the control of a P_trc_ promoter and a *lacI^Q1^* variant, which contains promoter mutations that increase the expression of *lac* repressor (Muller-hill *et al.*, 1968). We also made vectors lacking *rfaH* (pIA947) or containing substitutions in RfaH. Third, we constructed a reporter that carries a *Photorhabdus luminescens* (Winson *et al.*, 1998) *luxCDABE* operon under control of the P_BAD_ promoter/*araC* cassette (Guzman *et al.*, 1995) and the *ops* site (pGB83) and a control plasmid without the *ops* element (pGB63). This is a medium-copy-number plasmid and, since all natural RfaH targets are transcribed at very low levels, we did not use arabinose induction in our assays.

The *lux* operon encodes the luciferase (*luxA* and *luxB*) genes that oxidize FMN-H_2_ and a long-chain aliphatic aldehyde in the presence of O_2_ to yield a luminescence signal. The aldehyde is subsequently regenerated by a multi-enzyme reductase complex encoded by the *luxC*, *luxD* and *luxE* genes. The *lux* system is a sensitive (over at least five orders of magnitude) and simple bioreporter capable of autonomous light emission: it encodes all the components required to generate the bioluminescent signal, which is then directly measured in the cell culture.

In the absence of RfaH, expression of the *lux* operon was very low whether or not the *ops* element was present on the plasmid (0.3 and 1.2 units; Fig. 7B). In the presence of the episomally expressed WT RfaH, the reporter activity increased more than 700-fold when the *ops*^+^ template was used, and ∼20-fold with the control plasmid that lacks the *ops*. Thus, the observed activity was critically dependent on RfaH and was greatly enhanced by the *ops* element. Although the absence of *ops* did not completely eliminate the RfaH effect, the reporter activity was greatly reduced (from 217 to 26 units, approximately eightfold effect). The residual, *ops*-independent effect of RfaH could be due to (i) the ability of RfaH to bind to the TEC at other sites or (ii) the presence of an *ops*-like element somewhere on the reporter plasmid. Indeed, sequence analysis revealed an *ops*-like element in the *luxC* gene (GGCGGTAGAGca, marked as ops* in Fig. 7A, left). However, we found that substitutions in *luxC* that compromise the *ops* function *in vitro* did not alter either the *lux* reporter activity or its response to RfaH (unpublished). Interestingly, the RfaH effect *in vivo* is dramatically greater than its effect measured at any single site *in vitro*, consistent with the idea that RfaH effect is cumulative over many consecutive sites within the transcription unit and may include post-transcriptional events, such as recruitment of the translational machinery (Artsimovitch and Landick, 2002).

We next tested whether the isolated RfaH^N^ domain would function when expressed at a low level *in vivo*; overexpression of RfaH^N^ leads to its sequestration in the inclusion bodies. In agreement with our *in vitro* analysis (Belogurov *et al.*, 2007), RfaH^N^ was ∼1.5-fold more active than the WT protein and acted largely independently of *ops*; the inclusion of the *ops* element increased the RfaH^N^ activity only 1.3-fold.

The seven RfaH variants that displayed defects *in vitro* were also defective in *lux* operon expression; their activities ranged from 1% to ∼50% of the WT activity, and were increased 4- to 15-fold in the presence of *ops* (Fig. 7B). The most defective variant, Y54F, did not increase *lux* expression above background, consistent with its inability to reduce pausing at the *hisP* site (Fig. 6A). R73D, another strongly defective variant, displayed ∼20% of the WT activity and the strongest dependence on *ops* (a 15-fold difference), consistent with its pronounced defects in *ops*-binding and AP properties in the purified system. A somewhat higher activity of R73D as compared with Y54F could be due to a partial compensation of R73D defects at elevated RfaH concentration; R73A but not Y54F was rescued at 3 µM RfaH *in vitro* (Fig. 6). K10A, H20A and T66A variants retained ∼30–35% of the WT activity, whereas the mildly defective *in vitro* T72A and F51A variants were only approximately twofold less active than the WT RfaH.

Although the *in vitro* and *in vivo* phenotypes of the tested RfaH variants did not match perfectly, we did observe a correlation between their *in vitro* AP activity and the *in vivo* effects on *lux* expression. The observed differences are likely due in part to the nature of signals, which RfaH acts upon to increase the expression of the *lux* operon: while the hairpin-dependent *hisP* is an excellent model to study the mechanism of AP, most pause sites present on natural templates are short-lived pauses that control the overall rate of transcription and serve as precursors to termination. In addition, the presence of other cellular elongation factors or ongoing translation may affect RfaH function.

To ascertain that the *in vivo* defects of RfaH variants are not merely a result of their reduced expression or stability, we measured the cellular level of these proteins by Western analysis with polyclonal anti-RfaH antibodies (Belogurov *et al.*, 2009). These antibodies recognize epitopes within the RfaH^C^ domain (A. Sevostyanova, unpubl. obs.) and thus their binding should be unaffected by substitutions in RfaH^N^. We found that all variants containing single substitutions in RfaH^N^ were present at the same or a slightly higher level than the WT RfaH (Fig. 7C), suggesting that their defects are directly conferred by the substitutions; the level of expression of the isolated RfaH^N^ could not be measured by this assay.

## Discussion

RfaH is recruited to the TEC through specific contacts to the DNA and RNAP and remains bound to the enzyme until it completes the synthesis of the entire operon. While bound, RfaH increases the apparent rate of RNA synthesis on natural templates by suppressing pausing and reduces termination. In a purified system, all these activities are mediated by the N-terminal, RfaH^N^ domain. The data presented here suggest that RfaH^N^ contains three regions that mediate DNA recognition, retention on the RNAP throughout transcription, and AP modification. Below, we present the arguments in support of this hypothesis.

### A cluster of polar and charged residues mediates RfaH^N^ binding to DNA

Our previous studies indicate that RfaH directly and specifically binds to the NT DNA (Artsimovitch and Landick, 2002). Based on molecular modelling, we proposed that a polar region on RfaH^N^ interacts with *ops* (Belogurov *et al.*, 2007) in the RfaH/TEC complex. Importantly, this model was rather speculative: it was built using the structure of *T. thermophilus* RNAP, the only bacterial species for which high-resolution structures are available, but which differs from the *E. coli* enzyme in many surface features that may be essential for RfaH action. Furthermore, the path of the NT DNA was not constrained by any experimental data – none of the TEC structures currently available contains the NT DNA in the transcription bubble, and it is quite possible that both the *ops* sequence and the bound RfaH^N^ constrain its path on the RNAP surface.

The results of the mutational analysis that we carried out to evaluate this model (Fig. 5) are consistent with our predictions. Five residues that form a patch on the RfaH^N^ side opposite of the interdomain interface (Lys-10, Arg-16, His-20, Thr-72 and Arg-73) are required for RfaH-induced delay at *ops*, which we argue is due to persistent RfaH/DNA interactions. The effects of substitutions at these residues are unlikely to result from alterations in the protein structure (*Supporting information*); consistently, their defects were alleviated by increased RfaH concentration (Fig. 6). Importantly, the assay for RfaH/DNA interactions employed here is indirect, and additional lines of evidence (such as identification of *rfaH* suppressors of mutations in *ops* or a high-resolution model of RfaH/*ops* contacts) would be required to support this model.

RfaH^N^ does not have any recognizable DNA-binding motif, which is not surprising given that it binds to a rather unusual target: the single-stranded DNA strand exposed on, and interacting with, the surface of RNAP (Wang and Landick, 1997). The *ops* element spans 12 nt, among which 10 are highly conserved; however, the modelling predicts that only about four *ops* bases are available for direct contacts with RfaH^N^, too few to explain the high specificity of RfaH towards its targets observed *in vivo* (Belogurov *et al.*, 2009). The remaining *ops* bases likely mediate a conformational change in the TEC that is required for RfaH recruitment.

### A hydrophobic surface of the N-terminal domain mediates its binding to RNAP

The post-recruitment activity of RfaH depends on its persistent contacts to RNAP. We propose that these contacts are established between a hydrophobic patch on RfaH and the tip of the β′ CH. We reported that the deletion of the tip or substitutions of the Ile-290 or Ile-291 residues eliminated the RfaH ability to enhance elongation (Belogurov *et al.*, 2007). We also showed that RfaH Y8A and β′ I290R substitutions decreased the RfaH ability to compete with σ, presumably by destabilizing the RfaH/β′ CH contacts and allowing σ to bind to the β′ CH instead (Sevostyanova *et al.*, 2008). Here we show that substitutions of several residues in this region decrease the AP activity of RfaH (e.g. W4F and F56L; Fig. 6). The effect of these substitutions is alleviated by an increase in RfaH concentration, consistent with the reduced affinity of the altered proteins. Mutagenesis of *E. coli nusG*, *in vitro* analysis of NusG variants with substitutions of residues in the homologous patch (Mooney *et al.*, 2009b) and two-hybrid assays (Nickels, 2009) all suggest that NusG interacts with the β′ CH, supporting our assumption that RfaH^N^ and NusG^N^ bind to RNAP in a similar fashion (Belogurov *et al.*, 2009). Although alternative explanations are possible, we favour a model in which these hydrophobic residues make direct contacts to the β′ CH.

### The HTT motif as an AP module

The common activity of RfaH^N^ and NusG^N^ domains is to increase the rate of RNA chain elongation. This rate is limited by sequence-dependent signals that induce formation of an elemental pause state, in which the 3′ end of the nascent RNA is misaligned in the active site (Artsimovitch and Landick, 2000; Sydow *et al.*, 2009); this isomerization is thought to occur from a pre-translocated state (Toulokhonov *et al.*, 2007). Throughout elongation, RfaH likely maintains contacts with the DNA at the upstream part of the transcription bubble, where it would be poised to promote strand reannealing and thus translocation. Indeed, we showed that RfaH stabilizes the post-translocated TEC state and fails to act on pause-free templates and with pause-resistant RNAPs, suggesting that it prevents isomerization into the elemental pause (Svetlov *et al.*, 2007). In the simplest scenario, RfaH interactions with the β′ CH and the NT strand would be sufficient to induce forward translocation, thereby reducing pausing. In this scenario, RfaH should be able to act as long as it remains bound to the TEC.

**Fig. 8:**
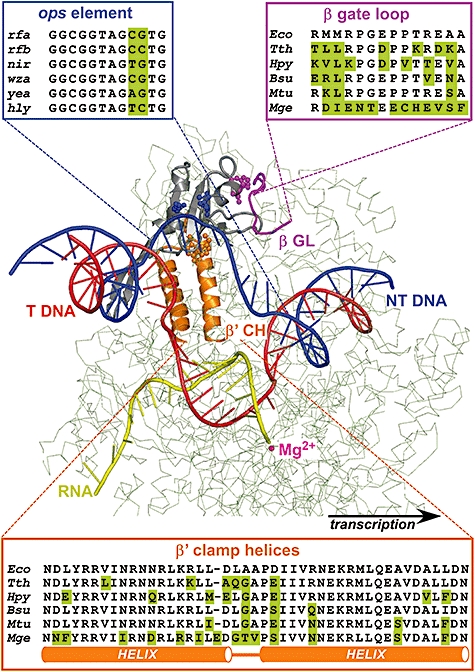
The functional contacts between RfaH and the TEC. We propose that RfaH function depends on three separate contacts with the NT DNA strand (blue), the β′ CH (orange), and the β GL (magenta). The side-chains of the RfaH residues that are proposed to make the key contacts with these elements are shown as spheres in the matching colours. The *ops* elements from selected operons that are associated with RfaH in MG1655 (Belogurov *et al.*, 2009) are conserved with the exception of the two highlighted bases. Sequences of the β′ CH and the β GL are highly and moderately conserved respectively; the residues that differ from the *E. coli* (*Eco*) sequence are shaded in light green. The abbreviations are: *Tth, Thermus thermophilus; Hpy, Helicobacter pylori; Bsu, Bacillus subtilis; Mtu, Mycobacterium tuberculosis; Mge, Mycoplasma genitalium*.

However, our data identify an additional HTT motif that is apparently required for AP by RfaH, but is distinct from the proposed DNA- and RNAP-binding motifs. The defects of substitutions in the HTT motif are not suppressed at high RfaH concentration (Fig. 6), suggesting that these changes are unlikely to cause the loss of affinity. In search for an alternative explanation, we re-examined the RfaH/TEC model (Belogurov *et al.*, 2007). We found that the HTT motif makes a hypothetical contact with the β gate loop (β GL, Fig. 8), a conserved loop that has been proposed to play a key role in the DNA loading in the course of the promoter complex formation (Vassylyev *et al.*, 2002). A large caveat of this modelling is that it has been performed with the *T. thermophilus* RNAP, in which the β GL sequence is quite distinct from that in the *E. coli* RNAP and which is not ‘designed’ to interact with RfaH, which is absent from many bacteria, including *Thermus*. However, if this contact can indeed be established in the *E. coli* TEC, it would offer a plausible hypothesis for the RfaH AP effects: the β GL belongs to a mobile domain that likely moves in concert with the β′ clamp during isomerization into a paused state (Toulokhonov *et al.*, 2007). During pausing and termination, the clamp is thought to partially open (Artsimovitch and Landick, 2000); RfaH could restrict the mobility of β and β′ parts of the clamp, thereby preventing its opening. Our recent data suggest that interactions between RfaH and the β GL are essential for RfaH function *in vivo* and *in vitro* but are not required for RfaH binding to the TEC (A. Sevostyanova *et al.*, manuscript in preparation).

### The functions of the RfaH domains

Our previous (Belogurov *et al.*, 2007; Svetlov *et al.*, 2007) and present (Figs 2 and 3) analyses show that RfaH^N^ acts *in vitro* at least as efficiently as the full-length protein. RfaH^N^ is both necessary and sufficient for binding to RNAP and to the *ops* DNA and mediates all the effects on elongation. However, the isolated RfaH^N^ is poorly soluble and lacks the key regulatory feature of RfaH, which distinguishes it from NusG: in contrast to the full-length protein, RfaH^N^ recruitment to the TEC is independent of the *ops* element.

RfaH^C^ has no discernible effects on transcription *in vitro* but plays several modulatory roles (Belogurov *et al.*, 2009). First, RfaH^C^ renders the full-length protein soluble by masking an extensive hydrophobic surface on RfaH^N^, which acts as an RNAP binding site. Second, RfaH^C^ restricts the RfaH action to a small number of *ops*-containing operons by preventing binding to RNAP except at the *ops* sites, where RfaH^N^ contacts with the DNA trigger domain dissociation. Third, RfaH^C^ may be engaged in cross-talk with the translation and secretion machineries; the last feature may be particularly important because RfaH-controlled operons are laterally acquired (and thus are likely poorly translated) and mediate synthesis of various extracytoplasmic molecules.

Our studies are in agreement with a recent analysis of the *E. coli* NusG domains (Mooney *et al.*, 2009b), which demonstrated that NusG^N^ is sufficient for the AP effect *in vitro* but fails to support the enhancement of Rho-dependent termination. NusG^C^ interacts with Rho and other partners: NusG is required for Nun-dependent termination and for the assembly of λ phage and *E. coli* rRNA anti-termination complexes. Different NusG^C^ substitutions eliminate the effect on either Rho- or Nun-dependent termination, suggesting that these proteins interact with distinct regions on NusG^C^ (Mooney *et al.*, 2009b).

RfaH has an opposite (and likely indirect) effect on Rho-dependent termination and does not interact with Rho; the most straightforward explanation is that RfaH^C^ structure is drastically different from that of NusG^C^ (essentially turned inside-out) and the Rho-interacting residues that are located on the surface of NusG^C^ are inaccessible in RfaH (Fig. 1). While it is possible that RfaH^C^ may refold into a β-barrel after recruitment, we argued that substitutions of residues in an RfaH-like protein that contact Rho in NusG (and whose identity is yet unknown) must have happened early after the ancestral *nusG* duplication to eliminate Rho binding, the key functional difference between the two paralogues (Belogurov *et al.*, 2009). We propose that RfaH^C^ may interact with other cellular proteins and may be essential for coupling transcription of RfaH-controlled operons to translation and finally to secretion. In support of this hypothesis, Bailey *et al.* (2000) reported that RfaH and *ops* nucleate the formation of a high-molecular-weight complex whose assembly requires RNAP and associated elongation factors, ribosomes, and the cytoplasmic membrane fraction.

C-terminal domains in eukaryotic NusG paralogues are more complex and likely mediate interactions with the components of the transcriptional and post-transcriptional complexes. For example, a plant-specific SPT5-like protein has a long carboxy-terminal extension that interacts with AGO4 to direct RNA-directed DNA methylation by polV (Bies-Etheve *et al.*, 2009) and transcriptional silencing of retrotransposons and repetitive elements.

## Experimental procedures

### Plasmids and strains

Plasmids used in this work are listed in Table S1. Sequences of all plasmid constructs were verified at the OSU PMGF centre and are available at our lab web site, http://www.osumicrobiology.org/homepages/artsimovitch/sequences/pIA_plasmids_list.htm. Disruption of the RfaH open reading frame (ORF) was carried out in the *E. coli* DH5α (λDE3) strain using TargeTron Gene Knockout System (Sigma-Aldrich, St Louis, MO, USA) according to the manufacturer's protocol. Briefly, RfaH ORF sequence was submitted to the proprietary search engine (http://www.sigma-genosys.com/targetron/) to identify potential target sites for intron insertion, and one of the top ranked targets (for intron insertion at position 21) was selected due to its proximity to the start codon. Intron insertion plasmid, pACD4K-C, was retargeted to this site on *rfaH* using PCR with a set of oligonucleotides generated according to the TargeTron algorithm. Disruption of *rfaH* by the retrohoming intron was induced by addition of IPTG to the culture transformed with the retargeted plasmid and application of the kanamycin selection. RfaH disruption was confirmed by genomic PCR and sequencing, followed by ‘curing’ the strain of the plasmid; the resulting Δ*rfaH* strain was named IA149.

### Proteins and reagents

Oligonucleotides were obtained from Integrated DNA Technologies (Coralville, IA, USA), NTPs and [α-^32^P]-NTPs were from GE Healthcare (Piscataway, NJ, USA), restriction and modification enzymes – from NEB (Ipswich, MA, USA), PCR reagents – from Roche (Indianapolis, IN, USA), other chemicals – from Sigma (St Louis, MO, USA) and Fisher (Pittsburgh, PA, USA). Plasmid DNAs and PCR products were purified using spin kits from Qiagen (Valencia, CA, USA) and Zymo Research (Orange, CA, USA). Rho protein was a gift from Rachel A. Mooney. The full-length RfaH variants, the RfaH^N^ domain and RNAP were purified as described in Belogurov *et al.* (2007).

### Halted complex formation

Linear templates for *in vitro* transcription were generated by PCR amplification. TECs were formed with 40 nM of linear DNA template and 50 nM RNAP holoenzyme in 20–100 µl of transcription buffer (20 mM Tris-chloride, 20 mM NaCl, 2 mM MgCl_2_, 14 mM 2-mercaptoethanol, 0.1 mM EDTA, 5% glycerol, pH 7.9). To make the elongation complexes halted after addition of G37 on pIA349 and pIA416 templates, transcription was initiated in the absence of UTP, with ApU at 150 µM, ATP and GTP at 2.5 µM, CTP at 1 µM, with ^32^P derived from [α-^32^P]-CTP (3000 Ci mmol^−1^). Halted complexes were formed for 15 min at 37°C and stored on ice prior to use.

### Single round pause assays

Halted [^32^P]-CTP labelled elongation complexes were prepared in 50 µl of transcription buffer. Elongation factors were added followed by 3 min incubation at 37°C. Transcription was restarted by addition of GTP to 15 µM, CTP, ATP and UTP to 150 µM, and rifapentin to 25 µg ml^−1^. Samples were removed at 10, 20, 40, 60, 90, 120, 180, 300, 600 and 1200 s and after a final 5 min incubation with 200 µM GTP (chase), and quenched by addition of an equal volume of STOP buffer (10 M urea, 50 mM EDTA, 45 mM Tris-borate; pH 8.3, 0.1% bromophenol blue, 0.1% xylene cyanol).

### Intrinsic termination assay at T*_hly_*

Halted complexes were prepared in 20 µl of transcription buffer with 25 nM of linear DNA pIA416 template and 60 nM of RNAP holoenzyme. Full-length RfaH, RfaH^N^ (or storage buffer) was added followed by 3 min incubation at 37°C. Elongation was restarted by addition of NTPs (10 µM UTP, 200 µM ATP, CTP, GTP) and rifapentin. Reactions were incubated at 37°C for 15 min and quenched as above.

### Rho-dependent termination assays

Halted complexes (A26) were prepared on pIA267 template in the absence of CTP in 30 µl of Rho buffer (40 mM Tris-HCl, 50 mM KCl, 5 mM MgCl_2_, 0.1 mM dithiothreitol, 3% glycerol, pH 7.9) supplemented with ApU at 150 µM, ATP and UTP at 2.5 µM, GTP at 1 µM, and 5 µCi of [α-^32^P]-GTP (3000 Ci mmol^−1^) during 15 min incubation at 37°C. Full-length RfaH, RfaH^N^ and NusG (or storage buffer) were added followed by 3 min incubation at 37°C. Transcription was restarted by addition of GTP to 15 µM, CTP, ATP and UTP to 150 µM, and rifapentin to 25 µg ml^−1^. Reactions were incubated at 37°C for 15 min and stopped as above.

### Sample analysis

Samples were heated for 2 min at 90°C and separated by electrophoresis in denaturing acrylamide (19:1) gels (7 M Urea, 0.5× TBE) of various concentrations (6–10%). RNA products were visualized and quantified using a PhosphorImager Storm 820 System (GE Healthcare), ImageQuant Software and Microsoft Excel. Kinetic analysis of pause assays is described in detail in the *Supporting information*.

### *In vivo* assays

A promoter-less *lux* reporter vector, pIA874, in which several unique restriction sites were engineered by site-directed mutagenesis, was constructed from pSB417 (Winson *et al.*, 1998). During these manipulations, we have realized that the actual sequence of pSB417 differs from the conceptual one, and thus completely sequenced the redesigned vector. To create a P_BAD_–*lux* fusion pGB063, a fragment containing the *araC* gene and the P_BAD_ promoter was PCR amplified from pBAD30 (Guzman *et al.*, 1995) and cloned into the NotI and XhoI sites of pIA874. The *ops*^+^ plasmid pGB083 was constructed by cloning a PCR fragment containing the *ops* element upstream of *rfbB* (amplified from *E. coli* DH5α genomic DNA) between the NheI and XhoI sites of pGB063. To test the RfaH effects *in trans*, a compatible plasmid (pIA947) was constructed by cloning a PCR fragment containing the PlacI^Q1^-*lacI* region from pIA249 (the *lacI^Q1^* variant was introduced into the primer) between the EagI and HindIII sites of pACYC184. To construct pIA957, an NdeI-HindIII fragment bearing wild-type RfaH was excised from pIA432 and cloned between the same sites of pIA947. Altered RfaH variants were recloned from pIA432-like plasmids listed in Table S1.

Plasmids carrying RfaH variants were co-transformed with a *lux* reporter vector (pGB083) into IA149 strain and plated on selective media (100 µg ml^−1^ carbenicillin, 50 µg ml^−1^ chloramphenicol). The single colonies were inoculated into 3 ml of Luria–Bertani (LB) media supplemented with antibiotics and incubated at 37°C with agitation. After 6 h of growth, cultures were diluted into fresh LB containing antibiotics and 0.1% glucose to an OD_600_ of ∼0.05 and allowed to grow for additional 6 h. Neither construct required induction (with IPTG and arabinose) since background expression of *rfaH* and *lux* operon was sufficient to generate the signal; this approach was chosen to mimic the very low expression levels of both *rfaH* and the operons it controls in *E. coli* (Belogurov *et al.*, 2009). Luminescence was measured in 200 µl aliquots in triplicates on FLUOstar OPTIMA plate reader (BMG LABTECH GmbH, Offenburg, Germany) and normalized by cell density. Results were analysed using Microsoft Excel.

### Western blotting

Derivatives of the *ΔrfaH* strain transformed with plasmids carrying RfaH variants or an empty vector were grown in the same conditions as for the Lux assay. Cell samples were collected by centrifugation and resuspended in 1 ml of Lysis buffer (50 mM Tris-HCl, 500 mM NaCl, 5% glycerol, 0.1 mM β-ME; pH 7.9) containing 0.1 mg ml^−1^ lysozyme. Cells were sonicated and extracts were cleared by centrifugation. Extract samples containing 22 µg of protein (as determined by Bradford assay) were loaded on a 10% SDS Bis-Tris gel (Invitrogen, Carlsbad, CA, USA). Protein transfer was performed in Tris-Glycine buffer, pH 8.3 containing 20% methanol onto Hybond™ ECL membrane (GE Healthcare, Piscataway, NJ, USA) at 300 mA for 2 h in a Mini Trans-Blot Cell (Bio-Rad Laboratories, Hercules, CA, USA). Blocking of non-specific sites was carried out overnight at 4°C in PBS-T buffer (1× PBS pH 7.5, 0.2% Tween-20) containing 5% non-fat dry milk. The membrane was incubated with rabbit polyclonal antibodies against RfaH (Belogurov *et al.*, 2009) diluted 1/4000 in PBS-T for 1 h with agitation at room temperature. After five washes with PBS-T, membrane was incubated with rabbit IgG for 1 h (1/10 000 dilution in PBS-T, obtained from GE Healthcare), washed again and exposed to ECL Plus detection reagents (GE Healthcare). Image was obtained using blue fluorescent mode on Storm-840 Phosphorimager (GE Healthcare).
