# Supplementary material for: Functional regions of the N-terminal domain of the antiterminator RfaH
Source: Mol Microbiol. 2010 Apr;76(2):286–301. doi: 10.1111/j.1365-2958.2010.07056.x (PMC2871177; doi:10.1111/j.1365-2958.2010.07056.x)
Supplement: Supplementary file 1 [file mmi0076-0286-SD1.pdf]

Supporting Information

Table S1. Plasmids and templates

Plasmid	Description	Ab^R	Reference /source
**RfaH overexpression vectors (pET derivatives):**<br>T7 gene 10 promoter-His_6-thrombin cleavage site-*rfaH*<br>*lacI*^{q}, ColEI *ori*, kanamycin resistance			
pIA238	*E. coli rfaH* between *Nde*I and *Hin*dIII sites of pET28b	Kn	(Artsimovitch & Landick, 2002a)
pIA432	silent *Bam*HI site in *rfaH* in pIA238	Kn	(Carter *et al.*, 2004)
pIA507	silent *Bam*HI and *Xho*I sites in *rfaH* in pIA432	Kn	this work
pIA674	RfaH W4A; site-directed mutagenesis of pIA238	Kn	this work
pIA675	RfaH Y5A ;site-directed mutagenesis of pIA238	Kn	this work
pIA676	RfaH W4F; site-directed mutagenesis of pIA238	Kn	this work
pIA677	RfaH Y8A; site-directed mutagenesis of pIA238	Kn	this work
pIA699	RfaH with a stop codon at 110 in pIA432	Kn	this work
pIA702	RfaH R40A; site-directed mutagenesis of pIA432	Kn	this work
pIA703	RfaH K37A; site-directed mutagenesis of pIA432	Kn	this work
pIA707	RfaH F51A; site-directed mutagenesis of pIA432	Kn	this work
pIA710	RfaH K10A; site-directed mutagenesis of pIA432	Kn	this work
pIA711	RfaH R11A; site-directed mutagenesis of pIA432	Kn	this work
pIA733	RfaH H94A; site-directed mutagenesis of pIA432	Kn	this work
pIA744	RfaH Q24A; site-directed mutagenesis of pIA507	Kn	this work
pIA756	RfaH K42A; site-directed mutagenesis of pIA432	Kn	this work
pIA757	RfaH H65A; site-directed mutagenesis of pIA432	Kn	this work
pIA758	RfaH T67A; site-directed mutagenesis of pIA432	Kn	this work
pIA760	RfaH H20A; site-directed mutagenesis of pIA766	Kn	this work

pIA761	RfaH E22A; site-directed mutagenesis of pIA766	Kn	this work
pIA763	RfaH E48A; site-directed mutagenesis of pIA238	Kn	this work
pIA764	RfaH T66A; site-directed mutagenesis of pIA507	Kn	this work
pIA766	silent *Sac*I, *Bam*HI and *Xho*I sites in *rfaH* in pIA507	Kn	this work
pIA767	RfaH Q2A; site-directed mutagenesis of pIA507	Kn	this work
pIA768	RfaH T68A; site-directed mutagenesis of pIA507	Kn	this work
pIA772	RfaH V63D; site-directed mutagenesis of pIA507	Kn	this work
pIA773	RfaH Q13A; site-directed mutagenesis of pIA507	Kn	this work
pIA783	RfaH E19A; site-directed mutagenesis of pIA507	Kn	this work
pGB009	RfaH R16A; site-directed mutagenesis of pIA238	Kn	this work
pGB012	RfaH Y54A; site-directed mutagenesis of pIA238	Kn	this work
pGB013	RfaH T72A; site-directed mutagenesis of pIA238	Kn	this work
pGB030	RfaH F56L; site-directed mutagenesis of pIA238	Kn	this work
pGB038	RfaH Y54F; site-directed mutagenesis of pIA238	Kn	this work
pAL16	RfaH R43A; site-directed mutagenesis of pIA432	Kn	this work
pVS61	RfaH R23A; site-directed mutagenesis of pIA432	Kn	this work
pVS62	RfaH R73D; site-directed mutagenesis of pIA432	Kn	this work
pVS66	RfaH R73A; site-directed mutagenesis of pIA432	Kn	this work
**RfaH expression vectors (pACYC derivatives):**<br>*lacI*^{Q1} promoter, P15A *ori*, *lac*I^{q}, chloramphenicol resistance			
pIA249	P_{lacI}-*lacI* and P_{trc}-*E. coli rfaH* in ptrc99	Amp	(Carter et al., 2004)
pIA947	P_{lacI}-*lacI*^{Q1} from pIA249 cloned between *Eag*I and *Hin*dIII sites of pACYC184	Cm	this work
pIA957	*rfaH* cloned between *Nde*I and *Hin*dIII sites of pIA947	Cm	this work
pIA1001	RfaH T66A in pIA957; recloned from pIA764	Cm	this work

pIA1002	RfaH K10A in pIA957; recloned from pIA710	Cm	this work
pIA1003	RfaH R73D in pIA957; recloned from pVS62	Cm	this work
pIA1004	RfaH H20A in pIA957; recloned from pIA760	Cm	this work
pIA1005	RfaH R16A in pIA957; recloned from pGB009	Cm	this work
pIA1006	RfaH Y54F in pIA957; recloned from pGB038	Cm	this work
pIA1007	RfaH T67A in pIA957; recloned from pIA758	Cm	this work
pIA1008	RfaH T72A in pIA957; recloned from pGB013	Cm	this work
pIA1009	RfaH F51A in pIA957; recloned from pIA707	Cm	this work
pIA1010	RfaH R43A in pIA957; recloned from pAL16	Cm	this work
pIA1058	$RfaH^N$ in pIA957; recloned from pIA699	Cm	this work
**Transcription templates**			
pIA267	λ P_R promoter–A26–*opsP*–λ_{tr1} Rho-dependent terminator transcription template	Amp	(Artsimovitch & Landick, 2002b)
pIA349	T7 A1 promoter–G37–*opsP*–*hisP* pause transcription template	Amp	(Artsimovitch & Landick, 2002b)
pIA416	T7 A1 promoter–G37–*opsP*–T_{hly} terminator transcription template	Amp	(Carter et al., 2004)
***Photorhabdus luminescens luxCDABE* reporter vectors [pSB417 (Winson *et al.*, 1998) derivatives]**: ColEI *ori*, ampicillin resistance			
pIA874	a polylinker cloned into pSB417 in place of the P_{lac} promoter, the entire plasmid was sequenced	Amp	this work
pGB063	*araC*-P_{BAD} promoter cassette from pBAD30 cloned into pIA874	Amp	this work
pGB083	an *ops* element from the *rfbB* gene cloned downstream from P_{BAD} in pGB083	Amp	this work

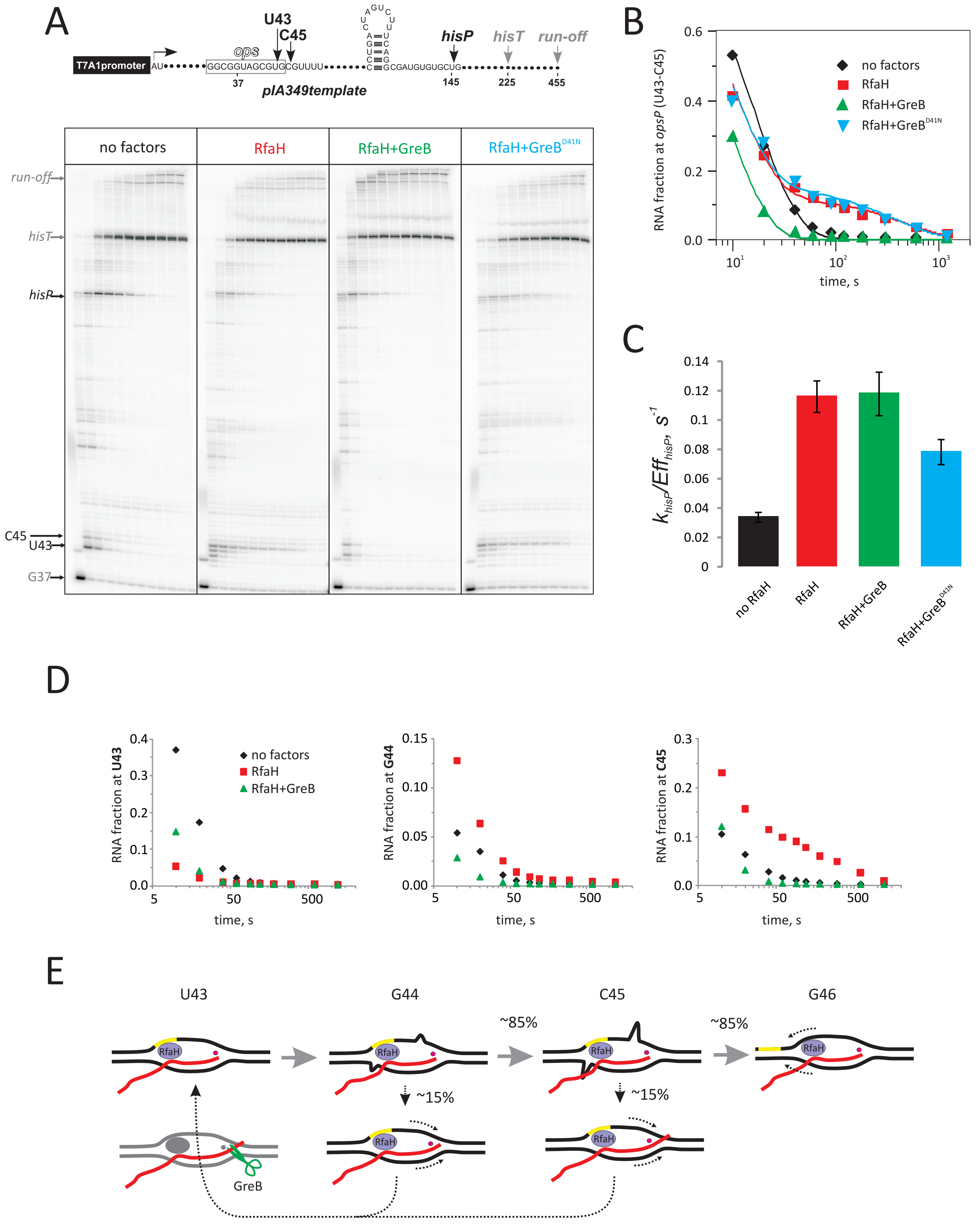
A
U43
C45
hisP
hisT
run-off
T7A1promoter
ops
pIA349template
37
145
225
455
no factors
RfaH
RfaH+GreB
RfaH+GreB D41N
run-off
hisT
hisP
C45
U43
G37
B
RNA fraction at opsP (U43-C45)
time, s
no factors
RfaH
RfaH+GreB
RfaH+GreB D41N
C
k_{hisP}/Eff_{hisP}, s^{-1}
no RfaH
RfaH
RfaH+GreB
RfaH+GreB D41N
D
RNA fraction at U43
RNA fraction at G44
RNA fraction at C45
time, s
no factors
RfaH
RfaH+GreB
E
U43
G44
C45
G46
~85%
~85%
~15%
~15%
RfaH
GreB

Fig. S1 Effects of GreB on pausing at the *ops* site

A. Top: A schematic representation of a linear template pIA349 with the *ops* element, the start site (+1), transcript end (run-off), the pause sites that occur after the addition U43 (*opsP1*), C45 (*opsP2*), and U145 nucleotides (the *hisP* pause), and the *hisT* terminator are indicated. Bottom: Halted radiolabeled G37 TECs (see Experimental procedures) were pre-incubated with RfaH at 50 nM or storage buffer for 5 min at 37 ^{o}C, and then challenged with rifapentin at 100 μg/ml and NTPs (10 μM GTP, 150 μM ATP, CTP, UTP). Where indicated, wild-type *E. coli* GreB or cleavage deficient variant GreB^{D41N} (Laptenko *et al.*, 2003) were included in transcription reactions at 500 nM. Aliquots were withdrawn at times ranging from 10 to 1200 sec and analyzed on a 8% denaturing gel.

B. The fraction of RNA at *opsP* (U43+G44+C45) as a function of time, quantified from the gel in (A). Wild-type GreB but not the cleavage deficient variant completely eliminates the slow escaping fraction of RNAP observed in the presence of RfaH.

C. Anti-pausing activity of RfaH at *hisP* expressed as k_{hisP}/Eff_{hisP} (see the next section) is not affected by GreB, suggesting that RfaH is properly recruited and retained with the TEC in the presence of GreB.

D. The fractions of RNAP retained at U43, G44 and C45 position as functions of time quantified from gel in (A). GreB dramatically reduces the RNAP fractions at C45 and G44 but increases the fraction at U43 compared to those observed in the presence of RfaH alone. Considering that GreB effects at pause sites are conventionally attributed to acceleration of RNA cleavage in backtracked TECs (Marr & Roberts, 2000), the data pattern above suggests that ~15% of RNAP is backtracked at positions C45 and G44 with the U43 nucleotide positioned in the active site.

E. The model of GreB and RfaH action at *opsP*. DNA strands and RNA are depicted as black and red lines, respectively. NT DNA nucleotides interacting with RfaH are colored yellow, catalytic magnesium ion is represented by a magenta sphere. RfaH (purple oval) is recruited to the TEC paused at U43 and maintains contacts with its binding site in NT DNA for two successive nucleotide addition cycles resulting in moderate scrunching of DNA. In ~15% of cases the scrunched state resolves by DNA reannealing downstream from the active site, thus generating a backtracked TEC with U43 in the active site. GreB (symbolically depicted as green scissors) regenerates active U43 TEC, which rapidly resumes elongation. Since the efficiencies of pauses at G44 and C45 are low, thus recovered TECs have a good chance to avoid getting trapped again, and GreB action ultimately results in rapid clearance of the *opsP* site as observed in (B).

Kinetic analysis of pausing at the *opsP* and *hisP* sites

To model RNAP pausing at *opsP,* we treated three successive RNA positions, U43 (the conserved essential pause, *opsP1*), G44 and C45 (the nonessential subpause, *opsP2*), as a single pause site. At the 5-s timepoint, 50-80% of RNAP was observed at such a combined *opsP*, and when allowed to vary, the fitted occupancy of *opsP* at zero time often approached 100%. Preliminary analysis demonstrated that RfaH and its variants affected the distribution of RNAP between U43, G44, C45 positions and the RNAP escape rate, but not the occupancy of the *opsP* extrapolated to zero time. Accordingly, for uniform analysis of all datasets we constrained the latter parameter to 100%.

The fraction of RNAP at the *opsP* site as a function of time *t* was described by equation 1a or 1b:

(1a) $opsP = e^{(-k_{fast}t)}$

(1b) $opsP = (1 - F_{slow})e^{(-k_{fast}t)} + F_{slow}e^{(-k_{slow}t)}$

For each dataset, both equations were fitted to data. The more complex equation 1b was employed for the final analysis when justified by the extra sum of squares F test with the significance level set to 0.05 (assays performed in the presence of the wild-type and most mutant RfaHs). The simpler equation 1a was used to analyze the data collected in the absence of transcription factors and some RfaH variants.

The equation 1b assumes that the *opsP* site is populated with 100% efficiency and generates two populations of RNAP, $1\text{-}F_{slow}$ and F_{slow} , which escape with the first order rate constants k_{fast} and k_{slow}, respectively. The model implies that *opsP* is populated instantaneously, which is a commonly used simplification. Considering that *opsP* is located just 8 bp downstream

of the halted complex, the expected time of RNAP arrival to this site is about 0.5 s at 15 nt/s. The influx of RNAP into *opsP* after 5 s is in most cases very small and does not need to be taken into account, permitting simple mathematical modeling of the process and precise determination of the fraction of slowly escaping RNAP and escape rate constants. The F_{slow} and k_{slow} parameters were also employed for accurate modeling of arrival of *opsP*-released RNAP at the *hisP* site (see below).

The fraction of RNAP arriving at the *hisP* site by the time *t* (designated as *ArrhisP*) was described by a differential equation 2a or 2b:

(2a) $ArrhisP' = \begin{cases} k_{arr}e^{(-k_{arr}(t-offset))}, & t-offset \geq 0 \\ 0 \quad , & t-offset < 0 \end{cases}$

(2b) $ArrhisP' = \begin{cases} (1-F_{slow})k_{arr}e^{(-k_{arr}(t-offset))} + F_{slow}k_{slow}e^{(-k_{slow}(t-offset))}, & t-offset \geq 0 \\ 0 \quad , & t-offset < 0 \end{cases}$

Both models assume that a non-zero minimal time represented by an *offset* parameter is required for RNAP to arrive at the *hisP* site located 108 bp downstream of the halted complex. Equation 2a was used in conjunction with equation 1a for the dataset where RNAP escape from *opsP* and, accordingly, arrival to *hisP* site followed the simple monoexponential function. Equation 2b was employed when escape from the *opsP* followed the biexponential function (equation 1b). According to equation 2b, RNAP populations that escaped from *opsP* with the first order rate constants k_{fast} and k_{slow} arrived at *hisP* with rate constants k_{arr} and k_{slow}, respectively. Thus, the fast escaping RNAP was slowed down by superimposition of the effects of multiple low-efficiency pauses upstream of the *hisP* site ($k_{fast} > k_{arr}$), whereas for the slow escaping fraction the release from the *opsP* site was the sole rate limiting step, and the arrival constant was the same as the *opsP* escape constant k_{slow}.

The diffusion of the RNAP front inevitably occurs, but with a relatively low sampling resolution of our assay it does not need to be taken into account to adequately describe the RNAP arrival at the *hisP* site. Importantly, the k_{slow} and k_{arr} parameters should not be confused with the mean rates of nucleotide addition, but rather represent the rate-limiting events that ultimately modulate the influx of RNAP at *hisP*.

The fraction of RNAP at *hisP* site as a function of time *t* was described by a differential equation 3:

$$(3)\quad hisP' = \begin{cases} Eff_{hisP} ArrhisP' - k_{hisP} hisP\,, & t - offset\ \geq 0 \\ 0\,, & t - offset\ < 0 \end{cases}$$

In this model, RNAP populates the *hisP* site with efficiency Eff_{hisP} and escapes with the first order rate constant k_{hisP}. The model implies that RNAP populations that were released from *opsP* rapidly (1-F_{slow}) and slowly (F_{slow}) differ only in the rate of arrival at *hisP,* which may be an oversimplification. However, our data do not allow for an independent inference of the slow fraction properties since (*i*) the slow fraction's escape rate from *opsP* was typically an order of magnitude lower than the *hisP* escape rate, making *opsP* the sole rate limiting step and (*ii*) the slow fraction usually accounted for less than a quarter of RNAP. In other words, slowly released RNAP never populated *hisP* to any measurable extent, making the determination of RNAP pausing propensity impossible. In addition, the influx and efflux rate constants (k_{arr} and k_{hisP}, respectively) were very similar at the *hisP* site, resulting in the apparent pausing efficiency (0.1-0.3) that is considerably lower than the fitted efficiency (0.5-0.8).

Importantly, under these conditions, the efficiency and pause escape rate parameters cannot be fully resolved because an increase/decrease in efficiency can be compensated by an increase/decrease in the escape rate constant. On the other hand, the k_{hisP}/Eff_{hisP} ratio could be

determined with high accuracy, and was very reproducible in repeated experiments. Accordingly, we analyzed the data with the modified model including k_{hisP}/Eff_{hisP} parameter instead of k_{hisP} and used k_{hisP}/Eff_{hisP} values for comparative evaluation of the AP activity of RfaH variants, as described in the Results section. For each dataset, the fractions of RNAP (1) at the *opsP* site; (2) arrived to *hisP*; and (3) at the *hisP* site were simultaneously fit to equations 1, 2 and 3, respectively, using numerical integration capabilities of Scientist 2.01 software [(Micromath; Bulirsch-Stoer method (Bulirsch & Stoer, 1991)].

Table S2. Predicted effects of selected substitutions in RfaH.

RfaH variant	$\Delta\Delta G_{calc}$* (kcal/mol)	$\Delta\Delta G_{LG}$ (kcal/mol)	$\Delta\Delta G_{es}$ (kcal/mol)	$\Delta\Delta G_{sa}$ (kcal/mol)	$-T^{\cdot}\Delta\Delta S$ (kcal/mol)	RMS (Å)
F56L	1.92	0.996	-0.242	1.21	-0.0478	1.291
H20A	2.55	1.95	-0.337	1.46	-0.529	1.630
R16A	-0.772	0.256	-0.677	0.332	-0.683	1.478
R43A	-0.5	-0.0833	-0.245	0.288	-0.46	1.361
K37A	1.39	1.49	-0.448	0.963	-0.614	1.331
E19A	-1.43	-0.634	-0.326	-0.0899	-0.384	1.569
H65A	2.62	2.02	0.063	1.19	-0.653	**2.162**
T66A	4.28	3.16	-0.0695	1.57	-0.384	1.537
T67A	1.98	1.42	0.0634	0.806	-0.306	**2.495**
Y54A	**3.66**	2.48	-0.727	2.51	-0.597	1.252
Y8A	**3.49**	2.86	-0.8	1.93	-0.505	1.382
T72A	1.93	1.65	-0.472	1.14	-0.395	1.144
W4A	**6.45**	4.6	-1.22	3.46	-0.392	1.797
W4F	**3.44**	1.83	0.0563	1.34	0.217	1.030
Y54F	-1.02	-0.67	-0.279	-0.155	0.0868	1.779
F51A	**4.69**	2.71	0.229	2.47	-0.71	1.749
H94A	2.84	1.95	-0.762	2.21	-0.562	**2.048**

*$\Delta\Delta G_{calc}=\alpha\Delta\Delta G_{es}+\beta\Delta\Delta G_{LG}+\gamma\Delta\Delta G_{sa}-\tau T^{\cdot}\Delta\Delta S$

To assess the impact of amino acid substitutions used throughout this work on the RfaH structure and stability, we utilized CONCOORD-PBSA molecular mechanics approach (Benedix *et al.*, 2009) using the *E. coli* RfaH structure (PDB ID 2OUG) as an input. http://ccpbsa.bioinformatik.uni-saarland.de/ccpbsa/index.php. For 12 variants, the predicted effects of substitutions (ΔΔG) were within 3 kcal/mol. Five substitutions (in bold) at the domain interface in the closed RfaH conformation, the only state for which structural information exists, were predicted to induce a greater decrease in stability (up to 6.54 kcal/mol for the W4A variant); however, the structure of the mutants is predicted to be largely intact, whereas the domain interface is destroyed after RfaH recruitment to the TEC, and the same region is thought

to instead bind to the ß' CH domain of RNAP (Belogurov *et al.*, 2007). Destabilization of the closed conformation might actually increase RfaH activity, but the detailed analysis of the effects of these substitutions would require a high-resolution experimental model of RfaH-TEC interactions. The predicted effects on stability did not correlate with a given mutant's anti-pausing activity, although variants with an increased stability tend to have increased or near-wild type levels of activity. The overall structure is also not predicted to change as a result of these substitutions: the total molecule RMS (root mean square) values deviated less than 2Å from the wild-type RfaH and they do not correlate with activity (Fig. S3). Predicted lowest energy structures for mutants fit quite well within WT structural ensemble (Fig. S2) further confirming that they belong to the same structural ensemble, accessible in solution. Flexibility changes were assessed by alignment of the sample structural ensembles for each mutant and the starting PDB structure and were found to be insignificant in the closed conformation of RfaH (data not shown). Altogether our modeling indicates that the impact of each single amino acid substitution on RfaH structure, flexibility and stability (ΔΔG) are rather moderate and are unlikely to account for majority of noted defects in mutants' activity.

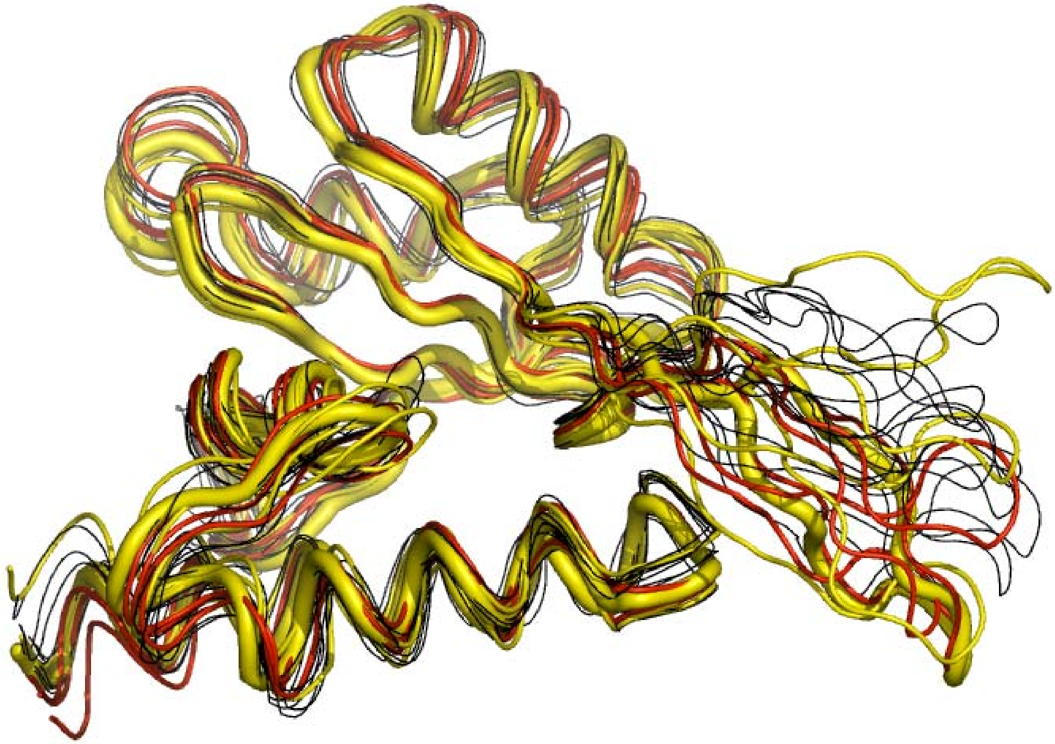

Fig. S2. Alignment of the wild-type and a representative set of "mutant" RfaH structures in context of the wild-type structural ensemble.

The tube radius for the wild-type ground state was set to 0.5, for "mutants" - to 0.15, for the higher energy WT structures - to 0.05. Cartoon is colored according to activity of RfaH variants in Fig. 6. Variants that possess near-wild type activity (F51A, H20A, K37A) are colored yellow; those displaying significant defects (R16A, T66A, W4F) are colored red; the wild-type structural ensemble is shown in black for visibility. The coordinates for alignment were obtained through CONCOORD-PBSA modeling, alignment and image were generated using PyMOL (DeLano, 2002).

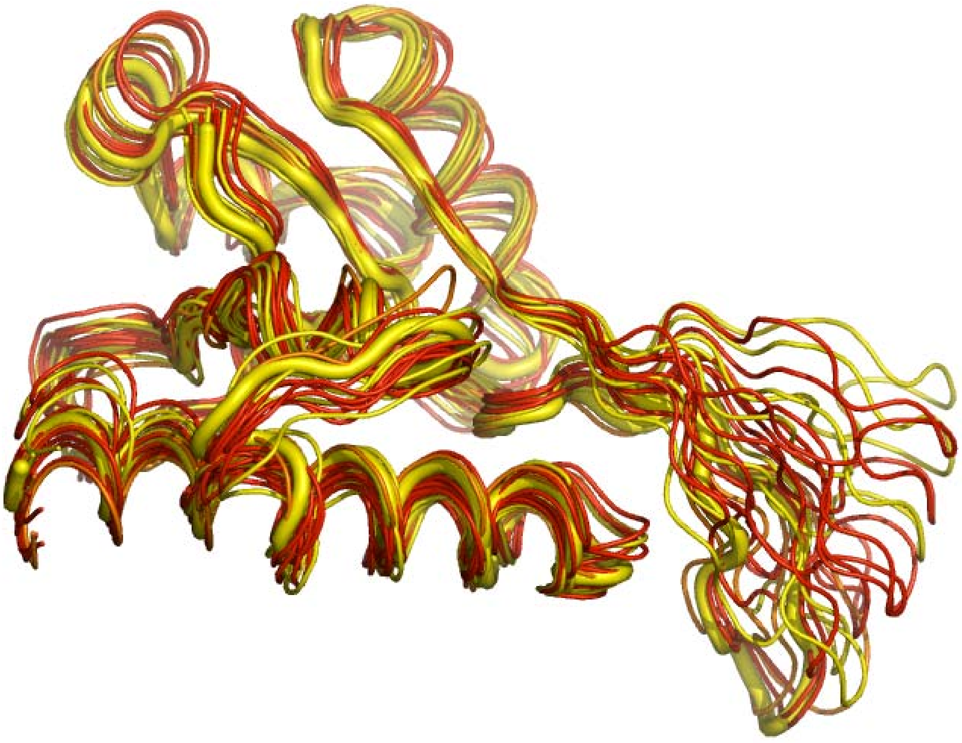

Fig. S3. Alignment of the wild-type and all “mutant” RfaH structures.

Tube cartoon representations (tube radius for WT was set to 0.5, for “mutants”to 0.15). Cartoon is colored according to RfaH activity in Fig. 6: the wild-type, K37A, E19A, H20A, F51A, H94A are shown in yellow; mildly defective R43A and T72A – in orange; very defective R16A, H65A, T66A, T67A, F56L, Y54A, Y8A, Y54F, W4F are shown in red. The coordinates for the alignment obtained through CONCOORD-PBSA modeling, alignment and image were generated using PyMOL.

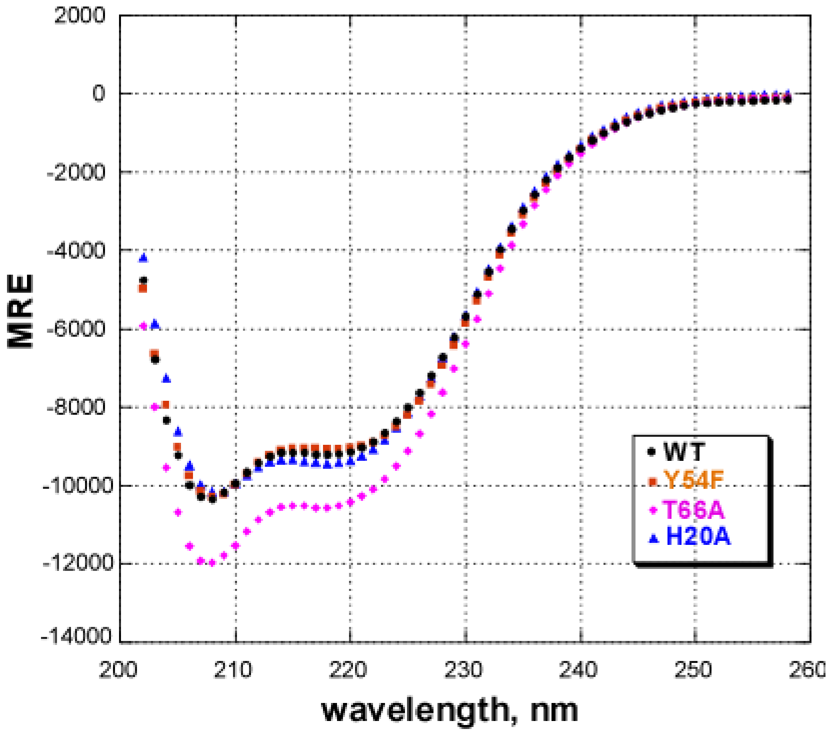

Fig. S4. Circular Dichroism (CD) spectra of selected RfaH variants does not reveal any major structural perturbations compared to the wild-type protein. Protein samples were dialyzed into 10mM Tris-HCl pH 8.0, 250mM NaCl, 1% glycerol and diluted to 19.5µM prior the experiment. CD spectra were recorded with an Aviv 62A DS CD spectrophotometer at 25 °C. Data were collected in a 1 mm quartz cuvette from 260 to 200 nm with a 1 nm step and 10 s averaging at each step. Mean residual ellipticity (MRE) was calculated as (q´100)/(0.1 cm´[P]´n) where q is raw ellipticity, [P] is protein concentration (µM) and n is the number of amino acid residues.

References

Artsimovitch, I. & R. Landick, (2002) RfaH stimulates chain elongation by bacterial transcription complexes after recruitment by the exposed nontemplate DNA strand. *Cell* **109**: 193-203.

Belogurov, G. A., M. N. Vassylyeva, V. Svetlov, S. Klyuyev, N. V. Grishin, D. G. Vassylyev & I. Artsimovitch, (2007) Structural basis for converting a general transcription factor into an operon-specific virulence regulator. *Mol Cell* **26**: 117-129.

Benedix, A., C. M. Becker, B. L. de Groot, A. Caflisch & R. A. Bockmann, (2009) Predicting free energy changes using structural ensembles. *Nat Methods* **6**: 3-4.

Bulirsch, R. & J. Stoer, (1991). In: Introduction to Numerical Analysis. New York: Springer-Verlag, pp.

Carter, H. D., V. Svetlov & I. Artsimovitch, (2004) Highly divergent RfaH orthologs from pathogenic proteobacteria can substitute for Escherichia coli RfaH both in vivo and in vitro. *J Bacteriol* **186**: 2829-2840.

DeLano, W. L., (2002) The PyMOL Molecular Graphics System. In. Palo Alto, CA: DeLano Scientific, , pp.

Laptenko, O., J. Lee, I. Lomakin & S. Borukhov, (2003) Transcript cleavage factors GreA and GreB act as transient catalytic components of RNA polymerase. *Embo J* **22**: 6322-6334.

Marr, M. T. & J. W. Roberts, (2000) Function of transcription cleavage factors GreA and GreB at a regulatory pause site. *Mol Cell* **6**: 1275-1285.

Winson, M. K., S. Swift, P. J. Hill, C. M. Sims, G. Griesmayr, B. W. Bycroft, P. Williams & G. S. A. B. Stewart, (1998) Engineering the luxCDABE genes from Photorhabdus luminescens to provide a bioluminescent reporter for constitutive and promoter probe plasmids and mini-Tn5 constructs. *FEMS Microbiology Letters* **163**: 193-202.
